# Women’s Health Consciousness and Attitude Towards Breast Cancer Screening in Rural Punjab, India: A Cross-Sectional Study in Ludhiana District

**DOI:** 10.7759/cureus.112572

**Published:** 2026-07-13

**Authors:** Arush E Michael, Abhay SK, Lovepreet Rai, Ashil SN, Akshaya T Krishnan, Himanshu Aggarwal, Clarence J Samuel

**Affiliations:** 1 Community Medicine, Christian Medical College Ludhiana, Ludhiana, IND; 2 Clinical Haematology, Christian Medical College Ludhiana, Ludhiana, IND; 3 Family and Community Medicine, Christian Medical College Ludhiana, Ludhiana, IND

**Keywords:** breast cancer screening, health consciousness, non-communicable diseases, rural health services, women’s health

## Abstract

Breast cancer has become more common in developing countries like India and is the most common cancer among women globally. Even though screening greatly increases survival, poor participation rates are still seen in rural areas. The purpose of this cross-sectional study, which was carried out in Lalton Kalan village, Punjab, was to provide insight into how sociodemographic traits affected the health consciousness and attitudes of 203 women who were 30 years of age and older regarding breast cancer screening. Data were collected using a pre-tested questionnaire that utilized the Health Consciousness Scale (HCS) and the Attitude Scale for Cancer Screening (ASCS). Both HCS and ASCS scores were found to be significantly impacted by age, education, marital status, occupation, and socioeconomic status. A positive correlation was established between screening attitudes and health consciousness (r = 0.441, t(201) = 6.96, p < 0.001). These results demonstrate the potential value of enhancing health education and incorporating breast cancer awareness into national health programs for disadvantaged rural populations.

## Introduction

Breast cancer is the second leading cause of death from malignancy in women, and its incidence continues to increase worldwide. It accounts for approximately one in four cancer cases and one in six cancer-related deaths among women [1]. Incidence rates of breast cancer are rising fast in transitioning countries in South America, Africa, and Asia. Dramatic changes in lifestyle, sociocultural, and built environments brought about by growing economies, along with an increase in the proportion of women in the industrial workforce, have affected the prevalence of breast cancer risk factors, including the postponement of childbearing, having fewer children, excess body weight, and physical inactivity, resulting in a convergence toward the risk factor profile of Western countries and narrowing international gaps in breast cancer morbidity [2]. Current trends point out that a higher proportion of the disease is occurring at a younger age in Indian women, as compared to the West [3]. The National Cancer Registry Program in India analyzed data from cancer registries for the period 1988-2013 to assess changes in cancer incidence. All population-based cancer registries have shown a significant increase in the trend of breast cancer [4].

Early detection of breast cancer is crucial in improving survival rates [5]. More than 80% of breast cancers in low- and middle-income countries are diagnosed in advanced stages. In conjunction with limited access to diagnostic and treatment services, this contributes to poor survival, as exemplified by the five-year survival of only 12% in Gambia and 46% in India, compared to over 85% in Western Europe and the United States [6,7]. According to the operational guidelines for cancer screening by the Government of India (GOI), breast cancer screening should be carried out for all women aged 30 and above through a clinical breast examination every five years [8]. Despite the guidelines and observed benefits of breast cancer screening, the recent National Family Health Survey (NFHS-5; 2019-2021) reported poor cancer screening coverage [9].

Health consciousness is the degree to which an individual is concerned about their health. It is a psychological construct that has been shown to influence health-related and preventive behaviors to act toward maintaining good health. The Health Consciousness Scale (HCS), developed by Gould, was employed in this study to assess this construct because it offers a comprehensive evaluation of health consciousness by assessing health self-consciousness, health alertness, health involvement, and health self-monitoring. It is especially appropriate for investigating general preventive health oriented in connection to breast cancer screening because of this more comprehensive evaluation [10]. Increasing awareness of healthy living in women will increase the early detection of breast cancer and survival rates by ensuring their regular participation in routine screening programs [11]. The study's primary objective was to assess health consciousness and attitudes toward breast cancer screening among rural women in Punjab. The study also aimed to examine the association between health consciousness and attitudes toward breast cancer screening, as well as selected sociodemographic characteristics.

## Materials and methods

Study design and setting

This study was conducted in a descriptive and cross-sectional design in Lalton Kalan village, Ludhiana District, Punjab, India, between April 2025 and June 2025. The population of the study included women aged 30 years and above residing in rural Punjab who were included in the breast cancer screening population according to the national breast cancer screening standards. The inclusion criteria were as follows: (a) women aged 30 years and above and (b) volunteers to participate in the study. The exclusion criteria were as follows: (a) having a history of breast cancer and (b) being a severely sick person.

Sample size and sampling technique

The sample of the study was determined according to the sampling method of the unknown population:

$n=\frac{Z^{2}\times p(1-p)}{d^{2}}$

where Z = 1.96 at the 95% confidence level, p = 0.50 (assumed prevalence), and d = 0.07 (absolute precision). Based on these assumptions, the minimum required sample size was calculated to be 196 participants. Since the research was not conducted within the framework of a screening program, no participant list was created. Participants were recruited using convenience sampling through household visits conducted in Lalton Kalan village. Data were collected through face-to-face interviews using a structured questionnaire during household visits. The consent forms were signed by the participants in person prior to data collection. Although the required sample size was calculated as 196, data from 203 participants were ultimately included to account for dropouts and incomplete data, as well as to enhance the robustness and reliability of the findings.

Study instruments

The study questionnaire included a demographic section along with the HCS and Attitude Scale for Cancer Screening (ASCS).

Demography section: The 11 questions in this section gathered socio-demographic characteristics from participants, such as age, residential area, educational level, marital status, and other relevant background details

HCS: This scale, which was developed by Gould [10] to measure the health consciousness of individuals, consists of nine items and four sub-dimensions (Health Self-Consciousness, Health Alertness, Health Involvement, and Health Self-Monitoring) that are assessed on a five-point, Likert-type scale ranging from ‘5: Strongly agree, 4: Agree, 3: Undecided, 2: Disagree, and 1: Strongly disagree.’ The total score ranges from 9 to 45, with a higher score indicating greater health consciousness. The scale has been repeatedly used in many studies, and its reliability and validity have been examined thoroughly [12,13]. To evaluate the internal consistency of the scale, Cronbach’s alpha coefficient, as indicated by Gould, was 0.947. In this study, it was found to be 0.78.

ASCS: The scale was developed by Öztürk et al. to measure attitudes toward cancer screening in adults aged 30-70 years [14]. The scale consists of 24 items in one dimension, which are graded on a five-point Likert-type scale ranging from ‘5: Strongly agree,' '4: Agree,' '3: Undecided,' '2: Disagree,' and '1: Strongly disagree.’ The lowest score that can be obtained from the scale is 24, and the highest score is 120. It is thought that as the scores approach 24, the participant has a negative attitude toward cancer screening, and when the score approaches 120, the participant has a positive attitude toward cancer screening. The scale does not have a specific cut-off point. While calculating the scale scores, 13 items (Items 9, 12, and 14-24) are reverse-coded. It is recommended to use the ‘6 - participant response’ formula for reverse coding. The Cronbach-alpha value was 0.95 in the study by Ozturk et al. In this study, it was found to be 0.96.

Statistical analyses

Data were analyzed using R version 4.4.1 (R Foundation for Statistical Computing, Vienna, Austria). Categorical variables were summarized as frequencies and percentages, while continuous scale scores were summarized as mean ± standard deviation (SD) and range. The internal consistency of the HCS and ASCS was assessed using the Cronbach alpha coefficient. The ASCS reverse-coded items (items 9, 12, and 14-24) were scored using the formula 6 - participant response before calculating the total score. Because HCS and ASCS were summed Likert-scale scores and were treated as approximately continuous summed scale scores, parametric tests were used for inferential analyses. Data normality was evaluated using the Kolmogorov-Smirnov test and visual inspection of quantile-quantile (Q-Q) plots, while the assumption of homogeneity of variance was assessed using Levene's test. Differences in mean HCS and ASCS scores across sociodemographic categories were assessed using one-way analysis of variance (ANOVA) for variables with more than two categories and the independent-samples Student t-test for binary comparisons. The F statistic with degrees of freedom was reported for ANOVA, and the t statistic with degrees of freedom was reported for t-tests. The association between HCS scores, HCS subdimension scores, and ASCS scores was assessed using Pearson correlation analysis, with the Pearson r, the corresponding t statistic, and p-value reported. All statistical tests were two-tailed, and a p-value < 0.05 was considered statistically significant. Categories with very small subgroup sizes were interpreted cautiously.

## Results

The majority of the participants belonged to the younger age groups of 30-39 (68, 33.5%) and 40-49 (57, 28.1%). Educational attainment varied, with the largest proportion having completed Class XII (45, 22.2%) or Class X (43, 21.2%), while 32 (15.8%) had no formal education and only 10 (4.9%) held a postgraduate or professional degree. Most of the participants (180, 88.7%) were homemakers, followed by employment in the private sector (6, 3%). Over half of the study participants (115, 56.7%) belonged to the lower-middle socio-economic status, with smaller proportions in the upper-lower (55, 27.1%) and upper-middle (24, 11.8%) categories, evaluated as per the modified Kuppuswamy scale. None of the participants belonged to the upper socio-economic status. Sikhism (178, 87.7%) was the predominant religion followed by most participants (Table 1).

**Table 1:** Sociodemographic characteristics of the study participants.

Characteristic	n (%)
Age group (years)	
30-39	68 (33.5)
40-49	57 (28.1)
50-59	27 (13.3)
60-69	30 (14.8)
≥70	21 (10.3)
​​​​​​Educational level	
No formal education	32 (15.8)
Less than Class VIII	21 (10.3)
Class VIII pass	34 (16.7)
Class X pass	43 (21.2)
Class XII pass	45 (22.2)
Graduate degree	18 (8.9)
Postgraduate/Professional degree	10 (4.9)
Marital status	
Unmarried	2 (1.0)
Married	182 (89.7)
Widow	19 (9.4)
Occupation	
Student	1 (0.5)
Homemaker	180 (88.7)
Public sector employee	2 (1.0)
Private sector employee	6 (3.0)
Self-employed	5 (2.5)
Unemployed	9 (4.4)
Religion	
Hindu	22 (10.8)
Christian	3 (1.5)
Sikh	178 (87.7)
Family history of breast cancer	
Yes	8 (3.9)
No	195 (96.1)
Socioeconomic status (Modified Kuppuswamy Scale)	
Upper middle	24 (11.8)
Lower middle	115 (56.7)
Upper lower	55 (27.1)
Lower	9 (4.4)

Age group was significantly associated with both HCS scores (F(4, 198) = 5.87; p < 0.001) and ASCS scores (F(4, 198) = 8.14; p < 0.001). Participants aged 70 years and above had the lowest mean scores in both domains. Education level was significantly associated with both HCS scores (F(6, 196) = 4.01; p < 0.001) and ASCS scores (F(6, 196) = 6.28; p < 0.001), with higher scores generally observed among women with higher educational attainment. Marital status was significantly associated with both HCS scores (F(2, 200) = 8.52; p < 0.001) and ASCS scores (F(2, 200) = 5.02; p = 0.007), with widowed participants showing lower mean scores than married participants. Occupation was significantly associated with both HCS scores (F(5, 197) = 3.31; p = 0.007) and ASCS scores (F(5, 197) = 4.87; p < 0.001). Religion was not significantly associated with either HCS scores (F(2, 200) = 0.68; p = 0.507) or ASCS scores (F(2, 200) = 0.80; p = 0.452). Socioeconomic status was significantly associated with HCS scores (F(3, 199) = 6.76; p < 0.001), whereas its association with ASCS scores was not statistically significant (F(3, 199) = 1.85; p = 0.140). Family history of breast cancer was significantly associated with HCS scores (t(201) = 2.77; p = 0.006) and ASCS scores (t(201) = 3.44; p < 0.001), although this finding should be interpreted cautiously because only eight participants reported a positive family history (Table 2).

**Table 2:** Comparison of mean health consciousness and attitude towards cancer screening scores according to sociodemographic characteristics. Data are presented as mean ± SD. Differences across variables with more than two categories were assessed using one-way analysis of variance (ANOVA), and the test statistic is reported as F with degrees of freedom (F(df1, df2)). Family history of breast cancer was compared using the independent-samples Student t-test, reported as t with degrees of freedom (t(df)). Statistical significance was set at p < 0.05. HCS: Health Consciousness Scale; ASCS: Attitude Scale for Cancer Screening; SD: standard deviation.

Characteristic	Category	n (%)	HCS score (mean ± SD)	HCS test statistic; p-value	ASCS score (mean ± SD)	ASCS test statistic; p-value
Age group (years)	30-39	68 (33.5)	38.4 ± 5.5	F(4, 198) = 5.87; p < 0.001	94.8 ± 19.9	F(4, 198) = 8.14; p < 0.001
	40-49	57 (28.1)	38.7 ± 4.7		90.9 ± 21.3	
	50-59	27 (13.3)	38.6 ± 6.1		96.6 ± 21.5	
	60-69	30 (14.8)	38.0 ± 5.7		80.6 ± 25.2	
	≥70	21 (10.3)	32.0 ± 8.6		68.0 ± 24.6	
Education level	No formal education	32 (15.8)	34.7 ± 7.7	F(6, 196) = 4.01; p < 0.001	73.8 ± 21.4	F(6, 196) = 6.28; p < 0.001
	Less than Class VIII	21 (10.3)	35.2 ± 7.2		82.7 ± 25.4	
	Class VIII	34 (16.7)	37.9 ± 5.3		82.4 ± 25.2	
	Class X	43 (21.2)	37.6 ± 5.1		95.8 ± 18.4	
	Class XII	45 (22.2)	39.5 ± 5.2		93.1 ± 21.2	
	Graduate	18 (8.9)	40.3 ± 5.1		103.8 ± 19.4	
	Postgraduate/Professional	10 (4.9)	41.3 ± 2.2		100.7 ± 20.5	
Marital status	Unmarried	2 (1.0)	35.0 ± 4.2	F(2, 200) = 8.52; p < 0.001	90.0 ± 24.0	F(2, 200) = 5.02; p = 0.007
	Married	182 (89.7)	38.4 ± 5.4		90.7 ± 22.6	
	Widowed	19 (9.4)	32.6 ± 9.0		73.3 ± 25.5	
Occupation	Student	1 (0.5)	32.0 ± NA	F(5, 197) = 3.31; p = 0.007	73.0 ± NA	F(5, 197) = 4.87; p < 0.001
	Homemaker	180 (88.7)	37.9 ± 5.7		88.9 ± 23.0	
	Public sector	2 (1.0)	39.5 ± 2.1		108.5 ± 2.1	
	Private sector	6 (3.0)	42.7 ± 2.9		116.8 ± 2.5	
	Self-employed	5 (2.5)	40.6 ± 2.2		102.0 ± 16.4	
	Unemployed	9 (4.4)	31.6 ± 11.4		64.1 ± 17.1	
Religion	Hindu	22 (10.8)	36.5 ± 7.0	F(2, 200) = 0.68; p = 0.507	92.0 ± 16.1	F(2, 200) = 0.80; p = 0.452
	Christian	3 (1.5)	39.7 ± 1.2		103.3 ± 20.2	
	Sikh	178 (87.7)	37.9 ± 6.0		88.5 ± 24.1	
Socioeconomic status	Upper middle	24 (11.8)	38.2 ± 5.1	t(201) = 2.77; p = 0.006	92.1 ± 22.8	t(201) = 3.44; p < 0.001
	Lower middle	115 (56.7)	38.9 ± 5.4		91.5 ± 23.7	
	Upper lower	55 (27.1)	36.3 ± 6.2	F(3, 199) = 6.76; p < 0.001	84.3 ± 22.6	F(3, 199) = 1.85; p = 0.140
	Lower	9 (4.4)	31.0 ± 9.5		79.3 ± 20.6	

The mean HCS score was 37.79 ± 6.05, with scores ranging from 12 to 45. The mean scores for the HCS subdimensions were 8.59 ± 1.46 for health alertness, 12.22 ± 2.08 for health self-consciousness, 8.45 ± 1.55 for health involvement, and 8.53 ± 1.69 for health self-monitoring. The mean ASCS score was 89.08 ± 23.31, with scores ranging from 31 to 120 (Table 3). A statistically significant positive correlation was observed between total HCS and ASCS scores using Pearson correlation analysis (r = 0.441, t(201) = 6.96, p < 0.001). All HCS subdimensions were also significantly positively correlated with ASCS scores. The strongest correlation was observed between the ASCS and the health self-consciousness subdimension (r = 0.499, t(201) = 8.16, p < 0.001) (Table 4).

**Table 3:** Descriptive statistics for the Health Consciousness Scale, its subdimensions, and the Attitude Scale for Cancer Screening. HCS: Health Consciousness Scale; ASCS: Attitude Scale for Cancer Screening; SD: Standard deviation.

Variable	Mean ± SD	Range
HCS	37.80 ± 6.04	12.00-45.00
HCS Health Alertness subdimension	8.59 ± 1.46	3.00-10.00
HCS Health Self-Consciousness subdimension	12.22 ± 2.08	4.00-15.00
HCS Health Involvement subdimension	8.45 ± 1.55	3.00-10.00
HCS Health Self-Monitoring subdimension	8.53 ± 1.69	2.00-10.00
ASCS	89.10 ± 23.30	31.00-120.00

**Table 4:** The correlation between the participants’ mean scores on the HCS and its subdimensions with the ASCS mean score. Correlations were assessed using Pearson correlation analysis. The test statistic is reported as t with 201 degrees of freedom. HCS: Health Consciousness Scale; ASCS: Attitude Scale for Cancer Screening.

	ASCS		
Variables	Pearson r	Test statistic	P-value
HCS	0.441	t(201) = 6.96	< 0.001
HCS Health Alertness subdimension	0.381	t(201) = 5.83	< 0.001
HCS Health Self-Consciousness subdimension	0.499	t(201) = 8.16	< 0.001
HCS Health Involvement subdimension	0.360	t(201) = 5.46	< 0.001
HCS Health Self-Monitoring subdimension	0.305	t(201) = 4.54	< 0.001

## Discussion

The outcomes of this study reveal substantial associations between sociodemographic characteristics, health consciousness, and attitudes toward breast cancer screening, offering significant insights for public health policies targeted at promoting early detection of breast cancer in underprivileged communities. Age had a major influence on both HCS and ASCS results. Younger participants (30-39 years) achieved higher scores in both domains than those aged 60 and above, who achieved the lowest scores. This age-related gradient is consistent with prior studies that demonstrated lower health-seeking behavior and cancer awareness among older women [15]. Multiple variables may contribute to this, including generational disparities in education, greater emotional and logistical barriers to seeking care, and deeply established sociocultural norms that undermine preventative health care among older women [16].

Education was additionally recognized as a key predictor of health consciousness and attitude. Women with postgraduate or professional degrees had the highest mean scores on both the HCS and ASCS, whereas those with no formal education or less than Class VIII education scored significantly lower. These findings corroborate the results of previous studies, which observed similar trends among India's population, correlating higher education with improved health literacy, screening compliance, and a readiness to seek medical care [17]. Education empowers women to comprehend disease processes and navigate the health care system with greater independence.

Participants from the lower-middle and upper-middle socioeconomic classes scored better on both scales than those from the lower class. This finding is consistent with a pattern documented in South Asia, which suggests that financial stability affects access to health information and autonomy in health-related decisions [18]. Self-employed women or those in the private sector demonstrated greater health consciousness and a positive attitude toward cancer screening when compared to homemakers or unemployed women. This indicates that women in formally employed positions may have more access to health promotion initiatives, financial resources, and decision-making autonomy [19]. These women may also be better socially connected, enabling the spread of health information. In our study, married women scored significantly higher than widowed and unmarried participants. Among widows, this could be attributed to a lack of social support, decreased financial autonomy, or psychological issues such as disengagement from self-care following the loss of their spouse. Similar findings were found in studies conducted in rural Nepal, where being widowed was associated with disregard for personal health due to cultural and systematic barriers to accessing healthcare [20].

The study demonstrated a moderate-to-high level of health consciousness among participants (mean HCS score = 37.79 ± 6.05), indicating that many women in rural areas are aware of their health. However, their moderately positive attitudes toward breast cancer screening (mean ASCS score = 89.10 ± 23.30) did not consistently convert into proactive screening behavior. A substantial positive link was identified between health consciousness and attitudes toward cancer screening. This suggests that health-conscious women are more likely to appreciate and encourage cancer screening activities. This association is consistent with previous studies, which suggest health-conscious individuals may be more likely to engage in preventative health measures, and that includes cancer screening programs [21]. Gould developed his multifaceted HCS, which incorporated awareness, involvement, self-monitoring, and attentiveness, all of which showed a positive correlation with screening attitudes in this study [10]. Notably, the ASCS and the health self-consciousness subdimension had the strongest correlation, highlighting the significance of personal reflection and health motivation in relation to preventive attitudes.

Future directions

Further research should be targeted toward larger and more multicentric populations. Rural and urban populations should be compared to increase the generalizability of findings. To ascertain whether improving health consciousness through focused educational and community-based awareness initiatives leads to long-lasting improvements in attitudes and actual participation in breast cancer screening, longitudinal and interventional studies are necessary. Subsequent research should also assess other barriers to screening behavior, such as cultural beliefs, access to healthcare, and family influence, and additionally examine how national screening programs and community health professionals could improve participation in breast cancer screening.

Limitations

The cross-sectional design does not permit causal inferences, and the use of convenience sampling from a single rural village may limit the generalizability of the findings beyond rural Punjab. Recall and social desirability bias cannot be ruled out because the data were self-reported. The questionnaires were not formally culturally adapted or psychometrically validated among Punjabi-speaking participants before use. Small sample sizes were used in certain subgroup analyses, and multivariable regression analysis was not used to adjust for potential confounding factors.

## Conclusions

This study's findings have various implications for rural India's public health practices. Health education campaigns could consider prioritizing older, less educated, and widowed women, who have been identified as demonstrating the lowest levels of health consciousness and poor attitudes toward cancer screening. Culturally appropriate health communication in local languages and accessible media formats such as folk theatre, community radio, and pictorial posters may help address existing knowledge gaps. Training community health workers in cancer-related health education and motivational interviewing techniques to effectively interact with women might. Furthermore, integrating breast cancer screening with existing national health programs may enhance opportunistic screening and health promotion. Interventions such as transportation subsidies, cash incentives, or fully subsidized diagnostic services can be helpful in addressing socioeconomic barriers. Finally, considering the influence of familial and patriarchal systems on women's health-seeking behavior in rural South Asia, including male family members in sensitization activities could generate more conducive environments for cancer screening participation.
